# Zinc-finger protein CXXC5 promotes breast carcinogenesis by regulating the TSC1/mTOR signaling pathway

**DOI:** 10.1016/j.jbc.2022.102812

**Published:** 2022-12-17

**Authors:** Wenjuan Wang, Zhaohan Zhang, Minghui Zhao, Yu Wang, Yuze Ge, Lin Shan

**Affiliations:** 1 Department of Biochemistry and Molecular Biology, School of Basic Medical Sciences, Capital Medical University, Beijing, China; 2 Beijing Key Laboratory of Cancer Invasion and Metastasis Research, School of Basic Medical Sciences, Capital Medical University, Beijing, China

**Keywords:** transcription factor CXXC5, transcription repression, mTOR signaling pathway, PD-L1-mediated immune suppression, vitamin, ChIP, chromatin immunoprecipitation, ChIP-Seq, chromatin immunoprecipitation sequencing, 4EBP1, eukaryotic translation initiation factor 4E–binding protein 1, GST, glutathione-S-transferase, H3ac, H3 acetylation, IP, immunoprecipitation, mTOR, mammalian target of rapamycin, NETN, NaCl, EDTA, Tris–HCl, Nonidet P-40, PD-L1, programmed cell death–ligand protein 1, qChIP, quantitative ChIP, qPCR, quantitative PCR, S6K, S6 kinase 1, TSC, tuberous sclerosis, TSC1, tuberous sclerosis complex subunit 1, TSC2, tuberous sclerosis complex subunit 2, WB, Western blot

## Abstract

CXXC5, a member of the CXXC family of zinc-finger proteins, is associated with numerous pathological processes. However, the pathophysiological function of CXXC5 has not been clearly established. Herein, we found that CXXC5 interacts with the CRL4B and NuRD complexes. Screening of transcriptional targets downstream of the CXXC5–CRL4B–NuRD complex by next-generation sequencing (chromatin immunoprecipitation sequencing) revealed that the complex regulates the transcriptional repression process of a cohort of genes, including TSC1 (tuberous sclerosis complex subunit 1), which play important roles in cell growth and mammalian target of rapamycin signaling pathway regulation, and whose abnormal regulation results in the activation of programmed cell death–ligand protein 1 (PD-L1). Intriguingly, CXXC5 expression increased after stimulation with vitamin B2 but decreased after vitamin D treatment. We also found that the CXXC5–CRL4B–NuRD complex promotes the proliferation of tumor cells *in vitro* and accelerates the growth of breast cancer *in vivo*. The expression of CXXC5, CUL4B, and MTA1 increased during the occurrence and development of breast cancer, and correspondingly, TSC1 expression decreased. Meanwhile, a high expression of CXXC5 was positively correlated with the histological grade of high malignancy and poor survival of patients. In conclusion, our study revealed that CXXC5-mediated TSC1 suppression activates the mammalian target of rapamycin pathway, reduces autophagic cell death, induces PD-L1-mediated immune suppression, and results in tumor development, shedding light on the mechanism of the pathophysiological function of CXXC5.

In mammals, to date, 12 members of the CXXC zinc-finger protein family have been identified. Unlike other members of this family, CXXC4 and CXXC5 function as transcription factors or epigenetic factors though binding to DNA but do not have any catalytic domains (1, 2). Remarkably, heterozygous *de novo* variants of the *CXXC5* gene are implicated in several childhood pathological processes, including immunodeficiency, sinusitis, and recurrent otitis media, as well as occasional pneumonia, suggesting a vital role for CXXC5 in development (3). In addition, *CXXC5* also acts as a tumor suppressor gene in acute myeloid leukemia, as low CXXC5 expression is correlated with upregulation of cell cycling genes and Wnt signaling (4). However, the role of CXXC5 depends on the specific type of cancer, as *CXXC5* has been reported to function as an oncogene in solid tumors, such as breast cancer, malignant melanoma, and papillary thyroid cancer (5). At the molecular level, CXXC5 acts as a transcriptional repressor. In CD8^+^ Th cells, CXXC5 was shown to associate with SUV39H1 to inhibit CD40 ligand expression (6). Under hypoxia, CXXC5 inhibits COX412 expression by interacting with an oxygen response element (7). Interestingly, contrary evidence proving that CXXC5 activates transcription has been reported in other studies (8, 9). Given these inconclusive results, the mechanistic action of CXXC5 in tumorigenesis requires further investigation.

Tuberous sclerosis (TSC) has been reported to be an inheritable disease resulting from mutations in either the TSC complex subunit 1 (*TSC1*) or 2 (*TSC2*) genes (10). In the cytoplasm, TSC1 forms a multimeric complex with TSC2 and TBC1D7 to play a classical role in inhibiting cell proliferation and migration by targeting the mammalian target of rapamycin (mTOR) signaling pathway, which plays a pivotal role in inhibiting autophagy in most eukaryotes (11, 12, 13). Abnormal activation of mTOR results in the activation of S6 kinase 1 (S6K) and inactivation of eukaryotic translation initiation factor 4E–binding protein 1 (4EBP1), leading to increased protein synthesis and cell proliferation (14, 15). Furthermore, dysfunction of the mTOR pathway participates in breast cancer tumorigenesis and the mechanism of resistance to endocrine therapy (16, 17, 18). Notably, inhibition of mTOR reduces the expression of programmed cell death–ligand protein 1 (PD-L1), which is known to confer cancer cells with the ability of immune evasion (19, 20); thus, the molecular basis of the dysregulation of the mTOR/autophagy pathway in breast cancer development requires further elucidation.

PD-L1, which is constitutively expressed on the surface of tumor cells, is known to bind to PD-1 on T cells, thereby aberrantly activating certain downstream oncogenic signaling pathways and ultimately impairing the antitumor function of T cells (21). Alternatively, PD-L1 can also be detected in the presence of T cell–derived cytokines, such as interferon gamma (22), during the process of adaptive immune resistance (23). The level of PD-L1 expression of breast cancer patients is positively correlated with poor prognosis (24). Altogether, this evidence underscores the significance of elucidating the molecular mechanisms of PD-L1 regulation in tumor cells, including epigenetic aspects.

Vitamins are known to have anticancer properties against many types of cancer, including breast cancer (25, 26, 27, 28, 29), which has drawn great attention for their potential therapeutic use (27, 28). Although this notion has been supported by observational epidemiological studies, these studies cannot provide a conclusive answer to the question of whether increasing intake of a certain vitamin reduces cancer risk; as such, further research and controlled human intervention trials are needed. Although research on the action mechanisms of vitamins have increased in recent years, revealing that vitamins affect various signaling pathways, including mTOR (30, 31), the functional link between vitamins and immune escape of tumors needs to be further explored to achieve optimal outcomes in immunotherapy.

In this research, we systematically investigated the pathophysiological functions as well as molecular basis of CXXC5 in tumorigenesis. We found that CXXC5 physically interacts with the CRL4B and NuRD complexes. We analyzed downstream genomic targets regulated by the CXXC5–CRL4B–NuRD complex and identified a set of genes, including *TSC1*, which can regulate the mTOR pathway. We also found that the repression of TSC1 by highly expressed CXXC5 leads to abnormal activation of the mTOR signaling pathway, thereby promoting cell proliferation *in vitro* and accelerating the growth of breast cancer *in vivo*, and is involved in the process of immune escape by regulating the expression of PD-L1. We revealed that CXXC5 expression in breast cancer is upregulated by vitamin B2 stimulation and that the expression level of CXXC5 is negatively correlated with vitamin D treatment and positively correlated with the degree of malignancy and poor prognosis of breast carcinomas.

## Results

### CXXC5 is physically associated with the CRL4B and NuRD complexes

**Figure 1:**
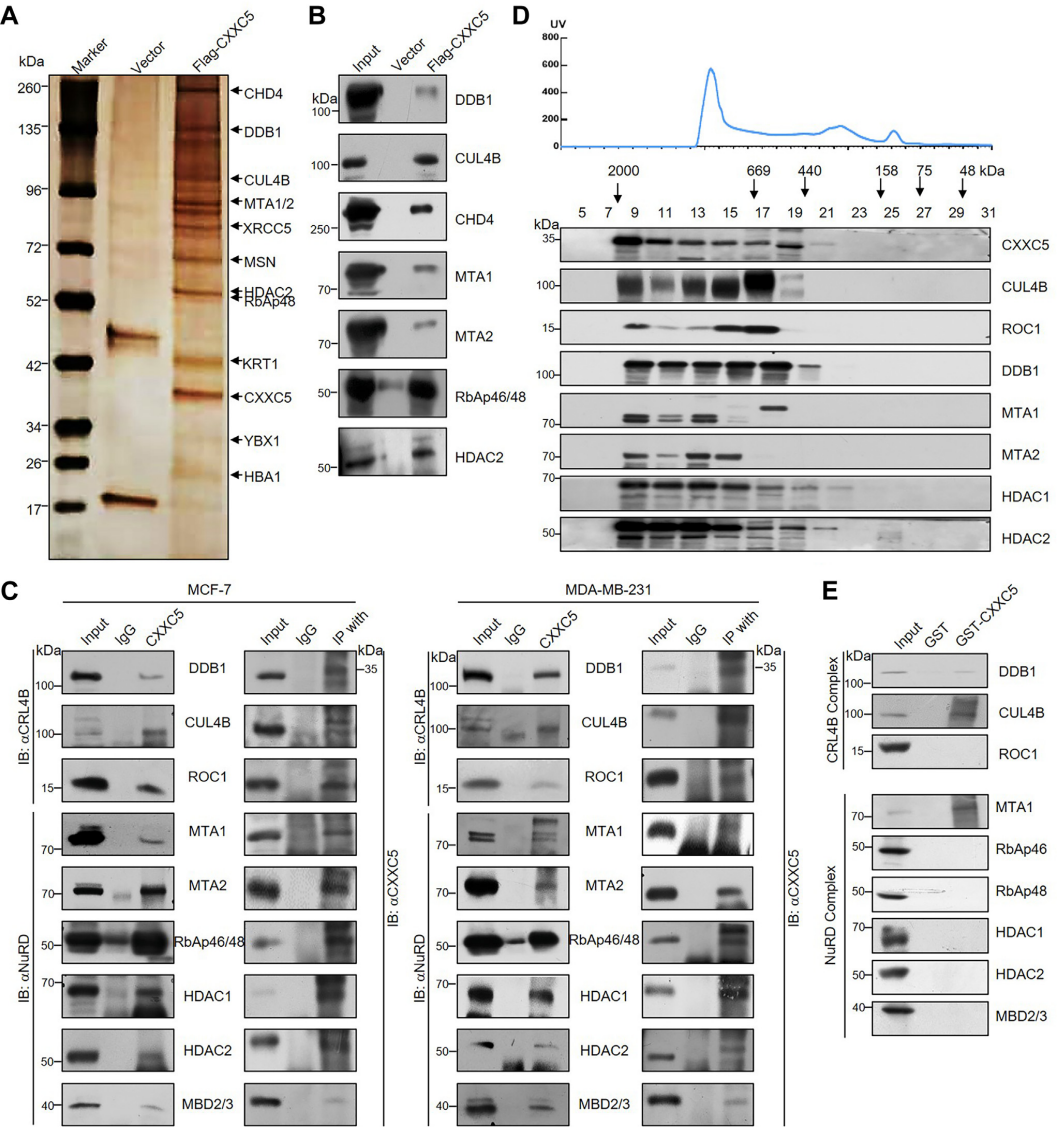
**CXXC5 is physically associated with the CRL4B and NuRD complexes.***A*, immunoaffinity purification and mass spectrometry detection of CXXC5-related protein complex. *B*, column-bound proteins were confirmed by Western blotting using antibodies against the specific proteins from the mass spectrometry results. *C*, coimmunoprecipitation (co-IP) was used to verify the interaction among CXXC5, the CRL4B complex, and the NuRD complex. Whole cell lysates from MCF-7 cells or MDA-MB-231 cells were used for IP with antibodies against CXXC5 followed by immunoblotting (IB) with antibodies against the indicated proteins or IP with antibodies against the indicated proteins followed by IB with antibodies against CXXC5. *D*, FPLC analysis. The *upper panel* shows the chromatographic elution profile, and the *lower panel* shows the results of Western blotting of the chromatographic portion containing antibodies against the complex component proteins. Each fraction is of equal volume and indicated by the elution position of the standard protein of known molecular weight (kilodalton). *E*, glutathione-*S*-transferase (GST) pull-down analysis was performed by *in vitro* reaction detection of the following specific proteins, which were obtained by bacterially purified GST recombinant expression and *in vitro* transcription/translation kits, respectively.

To elucidate the molecular basis of CXXC5 in breast carcinogenesis, we analyzed CXXC5-related proteins in tumor cells using MCF-7 cells as a model. By mass spectrometric analysis, we revealed that CXXC5 copurified with DDB1 and CUL4B (the components of the CRL4B complex), and with CHD4, MTA1, MTA2, HDAC2, and RbAp48 (the components of the NuRD complex) (Fig. 1*A*). Other proteins, such as XRCC5, MSN, and YBX1, were also detected. Table S1 lists the detailed data of mass spectrometry analysis. The components of the CXXC5-interacting complexes screened from the mass spectrometry results were further validated by Western blotting (WB) analysis using the column eluates with specific antibodies (Fig. 1*B*).

To further clarify the specific mechanism of action of CXXC5 in breast cancer development, we subsequently coimmunoprecipitated total protein extracts of MCF-7 and MDA-MB-231 cells with antibodies against CXXC5, and then immunoblotting with antibodies against DDB1, CUL4B, ROC1, MTA1, MTA2, RbAp46/48, HDAC1, HDAC2, or MBD2/3 was performed to confirm the interaction among CXXC5, the CRL4B complex, and the NuRD complex. The experimental results showed that all the aforementioned proteins could be efficiently coimmunoprecipitated with CXXC5 (Fig. 1*C*). In turn, immunoprecipitation (IP) with specific antibodies against either the CRL4B or the NuRD complex components followed by immunoblotting with antibodies against CXXC5 also showed that CXXC5 could be efficiently coimmunoprecipitated by all the components of both complexes (Fig. 1*C*). These results further confirm that CXXC5 interacts with the CRL4B and NuRD complexes.

To further verify the existence of CXXC5–CRL4B–NuRD complexes *in vivo*, we isolated and extracted the MCF-7 nucleoprotein, followed by FPLC with Superose 6 columns along with high-salt extraction and size-exclusion methods to separate the protein fractions. CXXC5 immunoreactivity was verified in chromatographic fractions from the Superpose 6 column, with a relatively symmetrical peak centered between approximately ∼669 and ∼2000 kDa, much larger than that of the monomeric protein of CXXC5 itself. Notably, the efflux pattern of CXXC5 was essentially consistent with the CRL4B complex protein patterns, such as those of CUL4B, ROC1, and DDB1, and with those of MTA1, MTA2, HDAC1, and HDAC2, which belong to the NuRD complex (Fig. 1*D*).

Next, to elucidate the molecular mode of action of the interaction between CXXC5 and related complexes, we purified CXXC5 protein by glutathione-*S*-transferase (GST) vector recombinants and in addition obtained complex components by *in vitro* transcription/translation kit for GST pull-down analysis. The results showed that CXXC5 interacted with DDB1, CUL4B, and MTA1 but not with the other protein components (Fig. 1*E*). Taken together, the aforementioned data confirm that there is a specific interaction among CXXC5, the CRL4B complex, and the NuRD complex.

### Genome-wide identification of transcriptional targets for the CXXC5–CRL4B–NuRD complex

**Figure 2:**
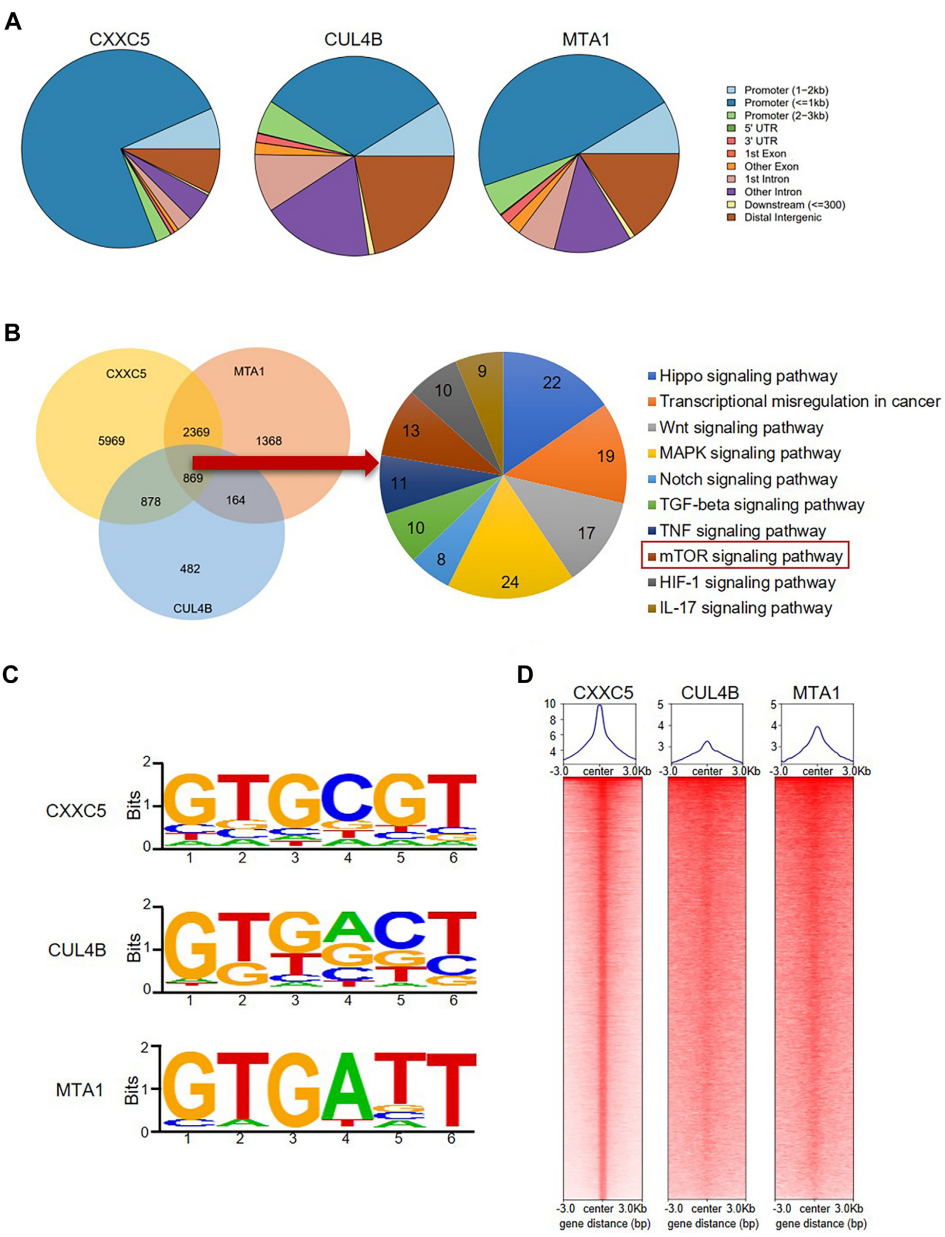
**Genome-wide identification of transcriptional targets for the CXXC5–CRL4B–NuRD complex.***A*, determination of the genomic distribution of CXXC5, CUL4B, and MTA1 binding sites by ChIP-Seq. *B*, a Venn diagram showing CXXC5, CUL4B, and MTA1 cobinding to promoters. After analysis, 869 overlapping target genes of CXXC5–CUL4B–MTA1 were obtained and clustered into functional groups. The detailed analysis results of ChIP-Seq have been summarized in Table S2. *C*, aanalysis of CXXC5-, CUL4B-, and MTA1-bound motifs using MEME suite. *D*, ChIP-Seq density heatmaps and profiles of CUL4B and MTA1 were analyzed against the CXXC5 binding sites as a background. ChIP-Seq, chromatin immunoprecipitation sequencing.

To elucidate the biological function of the interaction among CXXC5, the CRL4B complex, and the NuRD complex, we performed a genome-wide analysis of the transcriptional targets of CXXC5, CUL4B, and MTA1. To do this, we collected MCF-7 cells and performed chromatin immunoprecipitation sequencing (ChIP-Seq). Relevant DNA fragments pulled down by ChIP with CXXC5, CUL4B, or MTA1 antibodies were amplified, further labeled, and sequenced using the Illumina NovaSeq 6000 system. We then identified 30,244 CXXC5-specific binding peaks, 11,247 CUL4B-specific binding sequences, and 14,720 MTA1-specific binding sites using model-based analysis for ChIP-Seq, version 2 (MACS2) (Fig. 2*A*). Next, by crossanalyzing the ChIP-Seq data, we screened for overlapping DNA sequences, that is, targets of the CXXC5–CRL4B–NuRD complex (Fig. 2*B*). A total of 869 downstream genes directly bound by the CXXC5–CRL4B–NuRD complex were identified, including *TSC1*, which is well recognized for its role in the regulation of mTOR signaling and tumor suppression. Clearly, among CXXC5, CUL4B, and MTA1, genomic profiling revealed similar DNA-binding sequences (Fig. 2*C*). Comparing the characteristic enrichment of CXXC5, CUL4B, and MTA1 showed that CUL4B and MTA1 were significantly clustered near the CXXC5-binding sequences (Fig. 2*D*), strongly supporting the notion that CXXC5, the CRL4B complex, and the NuRD complex interact *in vitro* and *in vivo* and are functionally linked.

**Figure 3:**
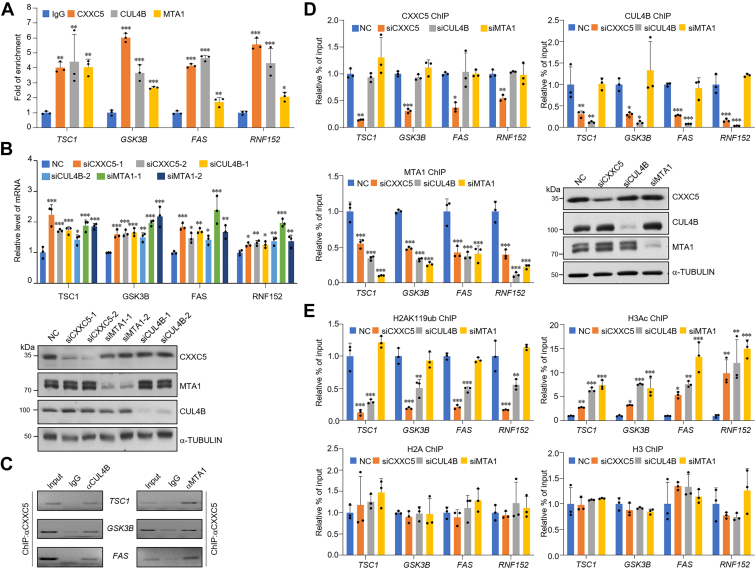
**Classification of downstream gene sets targeted by CXXC5-nucleated corepressor complexes.***A*, in MCF-7 cells, the ChIP-Seq results of the predicted genes with the indicated protein antibodies were verified by qChIP. The results show the calculation of fold change based on IgG. Each bar represents the mean ± SD from biological triplicate experiments. ∗*p* < 0.05, ∗∗*p* < 0.01, and ∗∗∗*p* < 0.001. *B*, MCF-7 cells were transfected with different groups of siRNAs to knockdown CXXC5, CUL4B, and MTA1, and the levels of representative downstream genes screened from ChIP-Seq were measured by RT–qPCR. Each bar represents the mean ± SD from biological triplicate experiments. ∗*p* < 0.05, ∗∗*p* < 0.01, and ∗∗∗*p* < 0.001. The knockdown efficiency of the proteins of interest was detected using Western blotting. *C*, ChIP/Re-ChIP experiments were performed for the promoters of the target genes with the proteins of interest antibodies. *D*, after transfection with the indicated siRNAs in MCF-7 cells, selected promoters were subjected to qChIP analysis in these cells using the indicated antibodies. Western blotting verified the knockdown efficiencies of CXXC5, CUL4B, and MTA1. Each bar represents the mean ± SD from biological triplicate experiments. ∗*p* < 0.05, ∗∗*p* < 0.01, and ∗∗∗*p* < 0.001. *E*, MCF-7 cells were transfected with siRNAs of the proteins of interest, and selected promoters were subjected to qChIP analysis in these cells using antibodies against histone modification, representing the function of the indicated proteins. Each bar represents the mean ± SD from biological triplicate experiments. ∗*p* < 0.05, ∗∗*p* < 0.01, and ∗∗∗*p* < 0.001. ChIP-Seq, chromatin immunoprecipitation sequencing; IgG, immunoglobulin G; qChIP, quantitative ChIP; qPCR, quantitative PCR.

Subsequently, we designed the promoter sequences representing target genes, including *TSC1*, *GSK3B*, *FAS*, and *RNF152*, and collected MCF-7 cells for quantitative ChIP (qChIP) assay to validate the ChIP-Seq results. The results showed that CXXC5, CUL4B, and MTA1 were highly enriched on the promoters of the selected targets (Fig. 3*A*). Moreover, knockdown of CXXC5, CUL4B, or MTA1 in MCF-7 cells and RT–quantitative PCR (qPCR) analysis of the selected genes were performed. The results showed that knockdown of the three proteins led to a significant increase in the expression of these downstream target genes to varying degrees (Fig. 3*B*). The efficiency of knockdown was detected using WB (Fig. 3*B*). To demonstrate that CXXC5, CUL4B, and MTA1 are present as a single protein complex on the promoters of the selected genes, we performed sequential ChIP, also known as ChIP/Re-ChIP, of *TSC1*, *GSK3B*, and *FAS*. During this experiment, we first performed IP of soluble chromatin with antibodies against CXXC5, and then re-IP was performed using the products of the first IP with CUL4B or MTA1 antibodies. The experimental results showed that the downstream gene promoters immunoprecipitated with CXXC5 antibodies could be re-IP with CUL4B or MTA1 antibodies (Fig. 3*C*). Together, these data confirm that *TSC1*, *GSK3B*, and *FAS* are targets of the CXXC5–CRL4B–NuRD complex and support that CXXC5, CUL4B, and MTA1 coexist as complexes on the promoters of these genes.

Next, to confirm that CXXC5 interacts with and recruits the CRL4B and NuRD complexes for the transcriptional repression of target genes, specific siRNAs were used to downregulate the expression of CXXC5, CUL4B, or MTA1 in MCF-7 cells. qChIP experiments in these cells demonstrated that loss of CXXC5 reduced the recruitment of CXXC5, CUL4B, and MTA1 to the promoters of *TSC1*, *GSK3B*, *FAS*, and *RNF152* (Fig. 3*D*). Meanwhile, although loss of CUL4B did not hinder the recruitment of CXXC5, it reduced the chromatin-binding ability of MTA1 (Fig. 3*D*). However, depletion of MTA1 did not affect CXXC5 and CUL4B promoter–binding ability (Fig. 3*D*).

Next, we investigated the epigenetic landscape shaped by CXXC5-directed recruitment of the CRL4B and NuRD complexes by qChIP. Upon knockdown of CXXC5, CUL4B, or MTA1 in MCF-7 cells with related siRNAs, the level of H2AK119ub on the promoters of *TSC1*, *GSK3B*, *FAS*, and *RNF152* was assessed as a function of the CRL4B complex activity, and pan-H3 acetylation (H3ac) levels on the same promoters were assessed as a function of NuRD complex activity. In CXXC5-deficient cells, the level of H2AK119ub on the promoters of targets was significantly reduced; at the same time, the H3ac level was significantly increased compared with that in the controls (Fig. 3*E*). Remarkably, consistent with the hypothesized recruitment scheme, CUL4B depletion led to not only reduced H2AK119ub levels but also increased H3ac levels (Fig. 3*E*). In MTA1-deficient cells, the level of H3ac was significantly increased on the promoters of targets, whereas the level of H2AK119ub was not altered (Fig. 3*E*). Taken together, the aforementioned data demonstrated that CXXC5 successively recruits CRL4B and the MTA1–NuRD complex to transcriptionally repress the expression of downstream target genes through epigenetic regulation.

### CXXC5 regulates mTOR signaling and impacts autophagy and protein synthesis by transcriptional repression of *TSC1*

**Figure 4:**
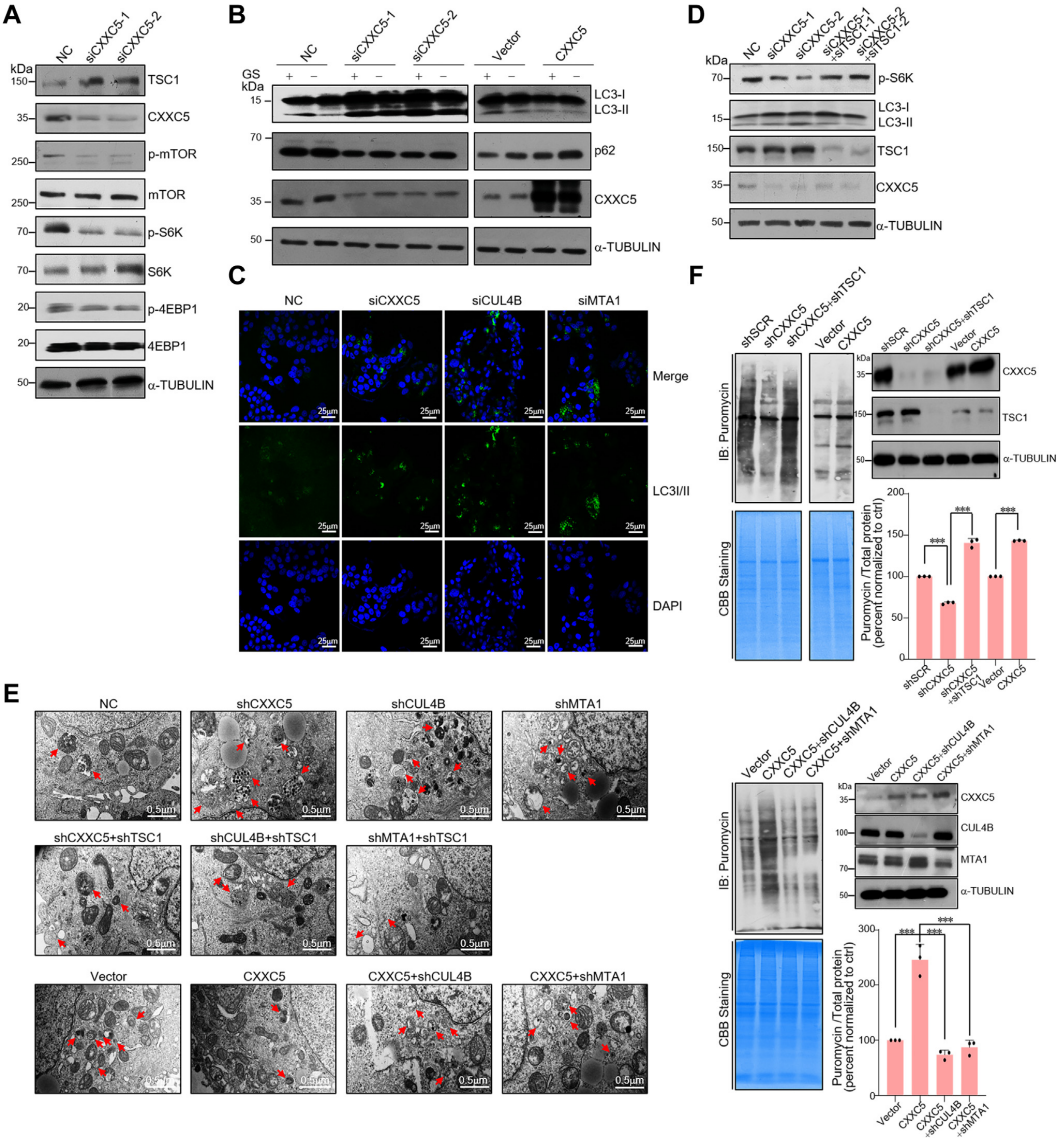
**CXXC5 regulates mTOR signaling and impacts autophagy and protein synthesis by transcriptional repression of *TSC1*** (Fig. S1). *A*, after transfection of different groups of CXXC5 siRNAs into MCF-7 cells, Western blotting (WB) was used to detect the indicated protein expression levels. *B*, MCF-7 cells with CXXC5 overexpression or knockdown were treated with or without 4 h glucose starvation (GS). Total cellular proteins were prepared and analyzed for the indicated proteins expression by WB. *C*, CXXC5, CUL4B, or MTA1 siRNAs were transfected into MCF-7 cells. The expression level of LC3I/II was detected by immunofluorescence microscopy (*green*). Nuclei were stained with DAPI (*blue*). *D*, the MCF-7 cells transfected with the indicated siRNAs were assayed for autophagy markers by WB. *E*, MCF-7 cells stably expressing the specific expression constructs and indicated shRNA lentiviruses were assayed for autophagosomes and autolysosomes by TEM. *F*, MCF-7 cells stably expressing the specific expression constructs and indicated shRNA lentiviruses were assessed for their ability for *de novo* protein synthesis using antipuromycin immunoblotting and pulsed with a final concentration of 1 μM puromycin for 30 min. Coomassie blue staining represented the total proteins. DAPI, 4′,6-diamidino-2-phenylindole; mTOR, mammalian target of rapamycin; TEM, transmission electron microscopy; TSC1, tuberous sclerosis complex subunit 1.

Aberrant regulation of *TSC1* occurs frequently in many tumorigenic processes (14, 15, 32), highlighting the possible critical role of precise regulation of TSC1 in breast tumor progression. To verify that *TSC1* could be transcriptionally repressed by the CXXC5–CRL4B–NuRD complex, CXXC5, CUL4B, or MTA1 was depleted in MCF-7 cells, and the level of TSC1 protein was detected using WB. Depletion of either CXXC5, CUL4B, or MTA1 resulted in an increased TSC1 expression level (Figs. 4*A* and S1*A*).

TSC1 participates in inhibiting the activation of the mTOR signaling pathway (13). Hence, we explored the specific biological function of the CXXC5–CRL4B–NuRD complex in modulating the mTOR signaling pathway. Knockdown of CXXC5, CUL4B, or MTA1 significantly reduced the phosphorylation levels of mTOR, S6K, and 4EBP1, indicating inactivation of the mTOR signaling pathway (Figs. 4*A* and S1*A*). Together, these data indicate that the CXXC5–CRL4B–NuRD complex activates the mTOR signaling pathway.

The mTOR signaling pathway is a suitable pharmacological target to manipulate autophagy (33). Thus, the effects of the CXXC5–CRL4B–NuRD complex on the level of phosphatidylethanolamine-conjugated LC3 proteins (LC3-II), a marker of autophagic flux, and p62, which accumulates when autophagy is inhibited (34), were detected. In MCF-7 cells with or without glucose starvation, knockdown of CXXC5, CUL4B, or MTA1 resulted in increased expression of LC3-II but a decreased level of p62 protein (Figs. 4*B* and S1*B*). In contrast, ectopic expression of CXXC5, CUL4B, or MTA1 suppressed glucose starvation–induced autophagy (Figs. 4*B* and S1*B*). In addition, knockdown of CXXC5, CUL4B, or MTA1 enhanced the number of anti-LC3I/II antibodies immunostained puncta in MCF-7 cells (Fig. 4*C*). The efficiency of knockdown was verified byWB (Fig. S1*C*). Consistent with the observation that the CXXC5–CRL4B–NuRD complex transcriptionally represses *TSC1*, depletion of TSC1 could partially rescue the changes in autophagy markers in cells with CXXC5, CUL4B, or MTA1 knockdown (Figs. 4*D* and S1*D*). Moreover, transmission electron microscopy revealed that depletion of CXXC5, CUL4B, or MTA1 in MCF-7 cells resulted in an abundant characteristic of autophagosomes and autolysosomes, both of which are markers of the autophagic process; in addition, concomitant knockdown of TSC1 in CXXC5-, CUL4B-, or MTA1-depleted MCF-7 cells was associated with a reversed trend in the number of autophagosomes and autolysosomes. Consistently, overexpression of CXXC5 in MCF-7 cells with glucose starvation resulted in scarce autophagosomes and autolysosomes, whereas knockdown of CUL4B or MTA1 attenuated the effect of CXXC5 on the alterations of the number of autophagosomes and autolysosomes (Fig. 4*E*). The efficiency of knockdown or overexpression was verified by WB (Fig. S1*E*). Taken together, these results support that CXXC5 and its related corepressor complexes are involved in the modulation of autophagy, and specifically, inhibit it by transcriptional repression of *TSC1*.

Since the mTOR signaling pathway is known to play a critical role in the regulation of protein synthesis (35), we assessed the potential effect of the CXXC5–CRL4B–NuRD complex on protein translation using a puromycin incorporation assay. The ability of *de novo* protein synthesis in CXXC5, CUL4B, or MTA1 knockdown cells was attenuated compared with that in the control group (Figs. 4*F* and S1*F*). Notably, shRNA knockdown of TSC1 rescued the decreased protein synthesis rates resulting from CXXC5, CUL4B, or MTA1 knockdown (Figs. 4*F* and S1*F*). Consistently, CXXC5, CUL4B, or MTA1 overexpression significantly increased the amount of synthesized proteins, and the increase in the amount of synthesized proteins associated with CXXC5 overexpression could be partially rescued by simultaneous depletion of CUL4B or MTA1 (Figs. 4*F* and S1*F*), highlighting the importance of the CXXC5–CRL4B–NuRD complex in modulating protein synthesis.

### CXXC5–CRL4B–NuRD promotes the proliferation of breast cancer cells *in vitro* and accelerates the growth of breast cancer *in vivo*

**Figure 5:**
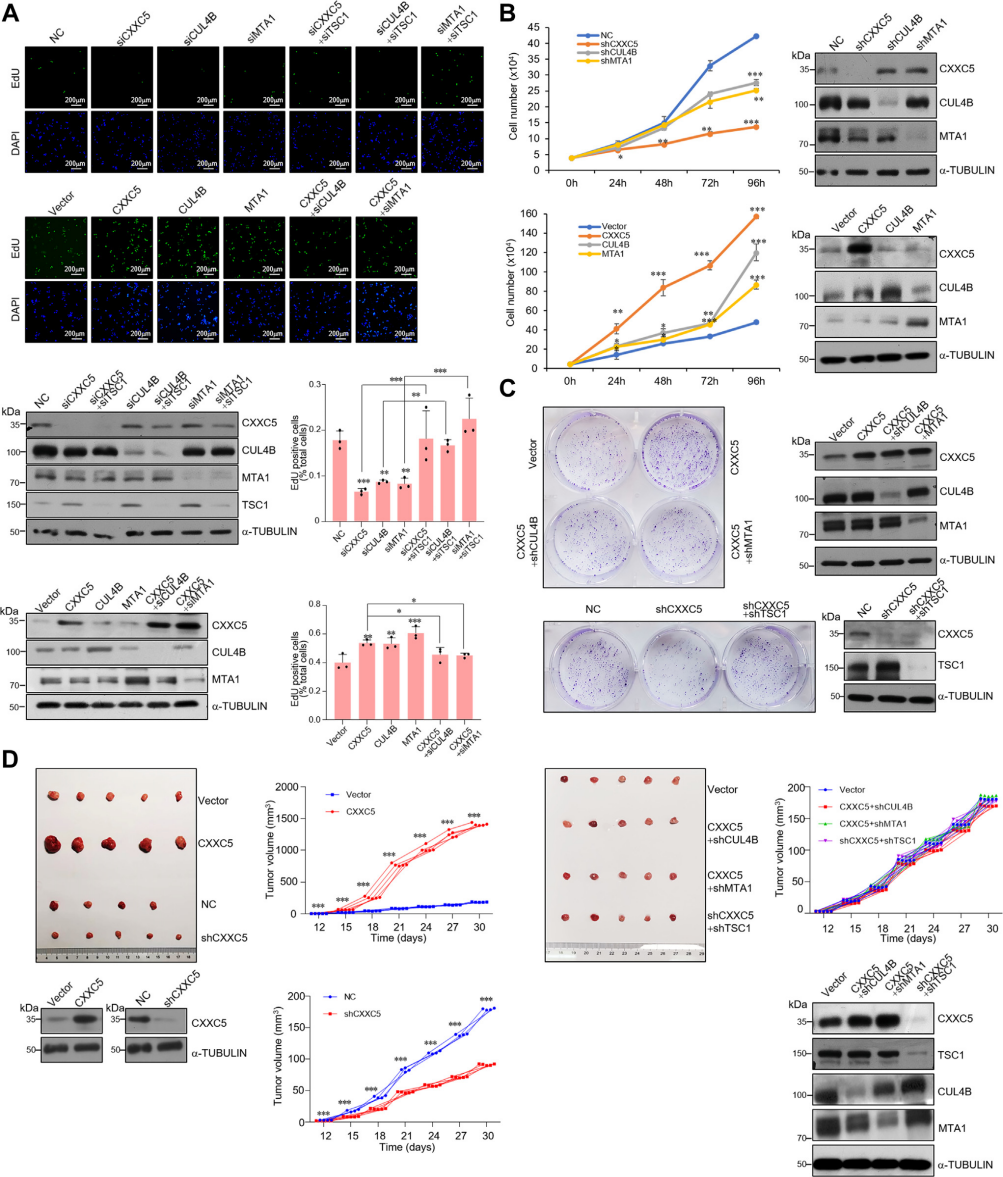
**CXXC5–CRL4B–NuRD promotes the proliferation of breast cancer cells *in vitro* and accelerates the growth of breast cancer *in vivo*.***A*, EdU assays were performed in MCF-7 cells transfected with the indicated siRNAs and the indicated expression constructs. Figures show representative images and statistical analysis. Each bar represents the mean ± SD from biological triplicate experiments. ∗*p* < 0.05, ∗∗*p* < 0.01, and ∗∗∗*p* < 0.001. Western blotting was used to detect the indicated protein expression levels. *B*, growth curve assays of lentivirus-infected MCF-7 cells carrying the knockdown shRNAs and indicated overexpressed constructs. Each bar represents the mean ± SD from biological triplicate experiments. ∗*p* < 0.05, ∗∗*p* < 0.01, and ∗∗∗*p* < 0.001. *C*, crystal violet staining was performed after culturing lentivirus-infected MCF-7 cells carrying the indicated overexpressed constructs and knockdown shRNAs for 14 days. Representative images are shown. *D*, MCF-7 tumors stably expressing shRNA and construction as indicated were transplanted onto ovariectomized athymic mice (n = 5). Representative tumors are shown, and tumor volumes were measured. ∗∗∗*p* < 0.001. The expression levels of the indicated proteins in these tumors were detected by Western blotting.

To elucidate the biological function of the CXXC5–CRL4B–NuRD complex in breast cancer progression, first, loss of function and gain of function of CXXC5, CUL4B, or MTA1 were performed in MCF-7 cells, and the effects of these treatments on cell proliferation were analyzed. Deletion of CXXC5, CUL4B, or MTA1 was associated with a significant reduction in the number of proliferating cells as confirmed by EdU cell proliferation assays (Fig. 5*A*). Meanwhile, loss of function of TSC1 partially alleviated the reduced cell proliferation induced by CXXC5, CUL4B, or MTA1 knockdown (Fig. 5*A*). Consistently, overexpression of either CXXC5, CUL4B, or MTA1 resulted in an increased number of proliferating cells (Fig. 5*A*), whereas knockdown of CUL4B or MTA1 in CXXC5-overexpressing MCF-7 cells resulted in diminished effects of CXXC5 overexpression (Fig. 5*A*). Growth curve measurements indicated that CXXC5, CUL4B, or MTA1 stable knockdown reduced the growth ability of breast cancer cells and that CXXC5, CUL4B, or MTA1 overexpression enhanced the growth of MCF-7 cells (Fig. 5*B*). Colony formation assays demonstrated that CXXC5 overexpression significantly increased the number of colonies, whereas CXXC5 knockdown reduced the number of colonies in MCF-7 cells (Fig. 5*C*). At the same time, loss of function of TSC1 partially alleviated the reduced number of colonies induced by CXXC5 knockdown; moreover, knockdown of CUL4B or MTA1 in CXXC5-overexpressing MCF-7 cells resulted in diminished effects of CXXC5 overexpression (Fig. 5*C*). Altogether, these data support the conclusion that CXXC5 promotes the proliferation of breast cancer cells by cooperating with the CRL4B and NuRD complexes to repress *TSC1*.

Next, we investigated the role of the CXXC5–CRL4B–NuRD complex in tumor development and progression *in vivo* by implanting MCF-7 cells that had been engineered to stably express empty vector, control shRNA, CXXC5, CXXC5 + shCUL4B, CXXC5 + shMTA1, shCXXC5, or shCXXC5 + shTSC1 onto the abdominal mammary fat pad of 6-week-old ovariectomized female athymic BALB/c mice implanted with E2 pellets (n = 5). We then monitored tumor growth over the next 4 weeks. The results showed that compared with the vector, CXXC5 overexpression was associated with a significant increase in the growth of primary MCF-7 tumors, whereas CXXC5 knockdown was accompanied by a decrease in the tumor growth (Fig. 5*D*). Notably, depletion of CUL4B or MTA1 in CXXC5-overexpressing tumors led to a diminished effect of CXXC5 overexpression; consistently, the decrease in tumor growth associated with CXXC5 knockdown could be rescued by the simultaneous silencing of TSC1 (Fig. 5*D*). Collectively, these experiments indicate that the CXXC5–CRL4B–NuRD (MTA1) complex has a significant effect on promoting tumor growth, and that it does so by repressing target genes, including TSC1.

### CXXC5 contributes to immune-escape ability of cancer cells and is bimodally regulated by vitamins

**Figure 6:**
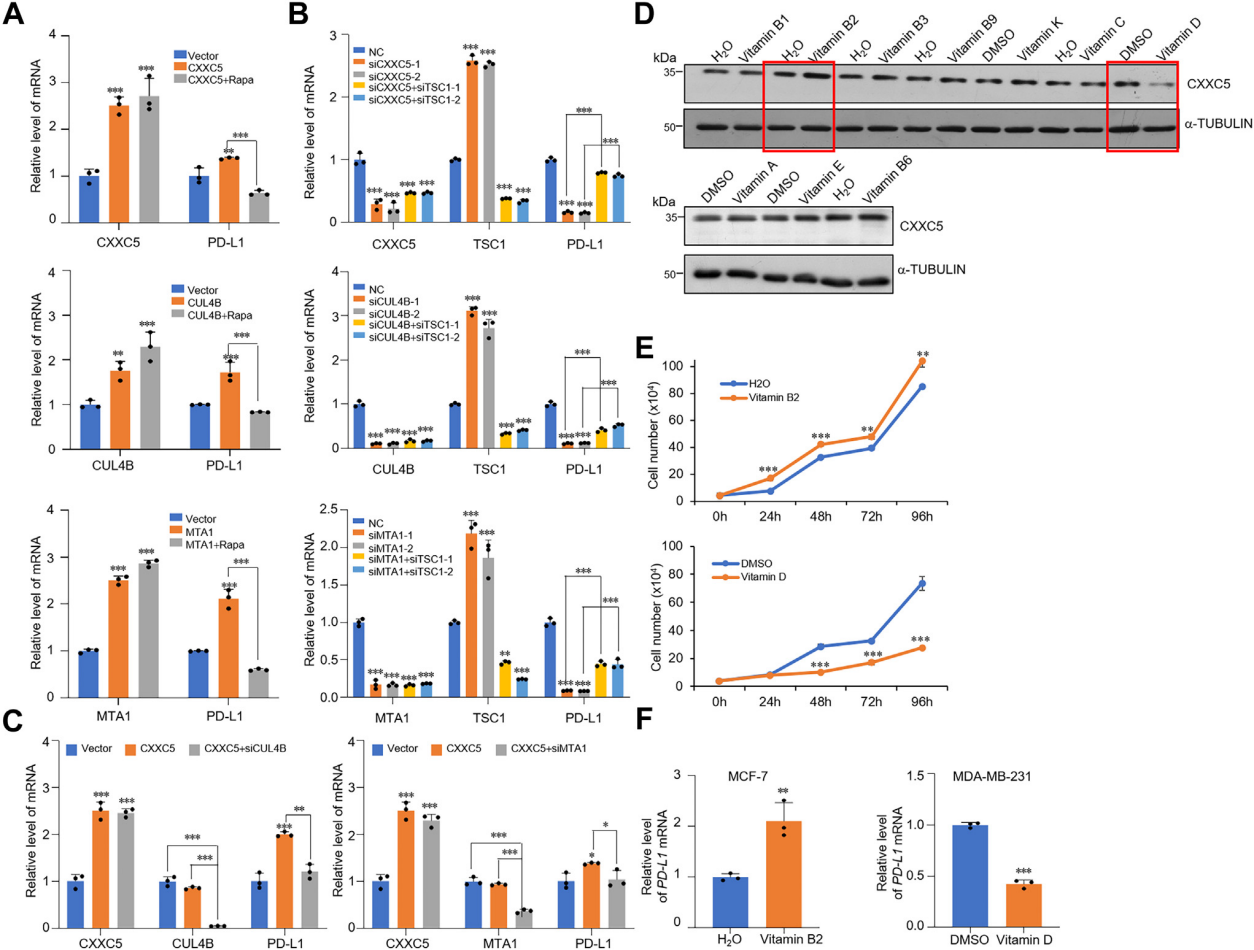
**CXXC5 contributes to immune escape ability of cancer cells and is bimodally regulated by vitamins.***A*, the level of PD-L1 mRNA in CXXC5, CUL4B, or MTA1 overexpression or/and 25 nM rapamycin-treated MDA-MB-231 cells was detected using RT–qPCR analysis. *B*, the mRNA level of PD-L1 in MDA-MB-231 cells transfected with control siRNA, CXXC5, CUL4B, MTA1 or/and co-ransfected with TSC1 siRNA was measured by RT–qPCR. *C*, the mRNA level of PD-L1 in CXXC5-overexpressing MDA-MB-231 cells transfected with CUL4B or MTA1 siRNA was measured by RT–qPCR. *D*, CXXC5 expression level in MCF-7 cells with different vitamin treatments was analyzed by Western blotting. *E*, growth curve assays of MCF-7 cells under different vitamin treatments. *F*, MCF-7 cells were treated with 266 nM vitamin B2, MDA-MB-231 cells were treated with 100 nM vitamin D, and the expression of PD-L1 was detected by RT–qPCR. PD-L1, programmed cell death–ligand protein 1; qPCR, quantitative PCR; TSC1, tuberous sclerosis complex subunit 1.

Blocking the PD-1–PD-L1 signaling pathway can effectively reactivate the function of T cells in lymphoma, serving as an effective therapy for various malignancies, including breast cancer (24, 36, 37). Interestingly, CXXC5 has been predicted to take part in the effectiveness of anti-PD-L1 treatment (38). To evaluate this, we selected MDA-MB-231 cells as the experimental model owing to their higher baseline expression of PD-L1. PD-L1 expression was assessed by overexpression or knockdown of CXXC5, CUL4B, or MTA1. RT–qPCR analysis demonstrated that CXXC5, CUL4B, or MTA1 overexpression led to increased mRNA level of PD-L1, whereas rapamycin treatment of CXXC5-, CUL4B-, or MTA1-overexpressing cells counteracted the changes in PD-L1 expression (Fig. 6*A*). Consistently, depletion of CXXC5, CUL4B, or MTA1 was related to a reduction in PD-L1 expression levels (Fig. 6*B*). Meanwhile, in CXXC5-, CUL4B-, or MTA1-depleted cells, co-knockdown of TSC1 reversed the expression level of PD-L1 (Fig. 6*B*). In addition, the synergetic effect of CXXC5 overexpression on PD-L1 expression appeared to be achieved through concerted action with the CRL4B and NuRD complexes because the effect diminished when CUL4B or MTA1 was concomitantly knock downed in MDA-MB-231 cells (Fig. 6*C*). This supports a role for CXXC5 in regulating the PD-L1 expression and suggests that it does so through its association with the CRL4B and NuRD complexes and suppression of downstream target genes including TSC1.

Vitamins are involved in numerous cellular biological processes, such as cell proliferation, metabolism, and aging (39, 40, 41). Dysregulation of all these processes has been associated with mTOR activation and carcinogenesis (25, 26, 27, 28, 29, 42). Based on this, we speculated that vitamins could influence the expression of CXXC5. To confirm this conjecture, we treated MCF-7 cells with various types of vitamins and determined the expression level of CXXC5 after treatment using WB. The results indicated that CXXC5 expression increased after stimulation with vitamin B2 but decreased after vitamin D treatment (Fig. 6*D*). Consistently, growth curve measurements showed that stimulation with vitamin B2 promoted the proliferation of cells, whereas vitamin D treatment decreased the growth rate (Fig. 6*E*). Moreover, consistent with the expected functional link between CXXC5 and PD-L1, RT–qPCR analysis showed increased PD-L1 expression in MCF-7 cells with lower cell surface expression at baseline following vitamin B2 treatment. In contrast, vitamin D treatment of MDA-MB-231 cells significantly reduced the level of PD-L1 (Fig. 6*F*). Overall, these results indicate that CXXC5 expression is upregulated by vitamin B2 and downregulated by vitamin D treatment and contributes to immune escape of breast cancer cells.

### CXXC5 expression is elevated in breast cancer and positively correlates with tumor grade, corresponding to the low expression of TSC1

**Figure 7:**
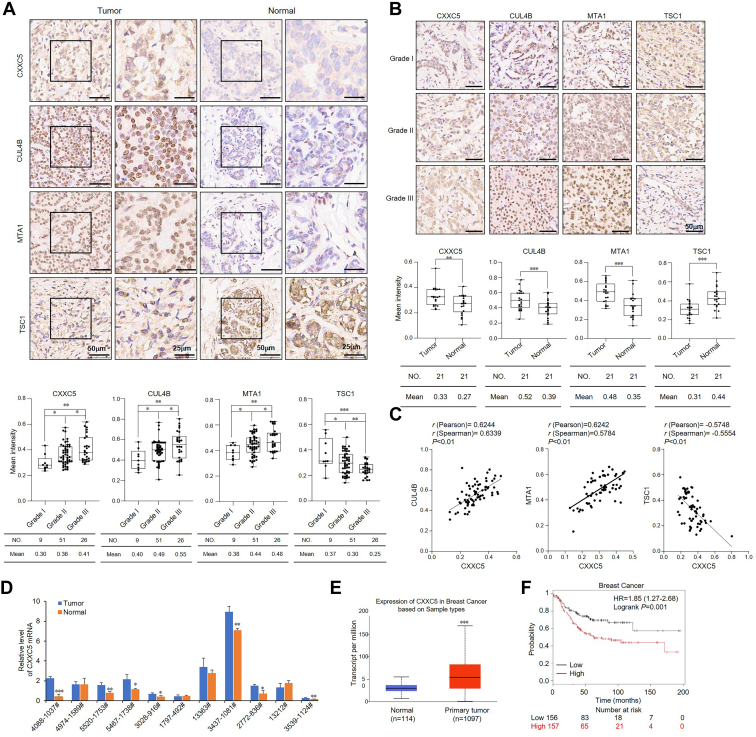
**CXXC5 expression is elevated in breast cancer and positively correlated with tumor grade, corresponding to the low expression of TSC1.***A*, immunohistochemical staining for CXXC5, CUL4B, MTA1, and TSC1 in tissue arrays, including 21 grade II breast cancer samples and adjacent normal breast tissues. The representative images are presented, and positively stained nuclei were detected. The Image-Pro Plus software was used to calculate the mean staining intensity. *B*, immunohistochemical staining of CXXC5, CUL4B, MTA1, and TSC1 expression in tissue arrays, including 86 specimens of breast carcinoma, including grades I, II, and III. Representative images are presented, and positively stained nuclei were counted. Using Image-Pro Plus software, the mean staining intensity was calculated. The *middle*, *upper*, and *lower three lines* of the boxplot represent the mean, upper, and lower quartiles, respectively, of the relative intensity calculated from CXXC5, CUL4B, MTA1, and TSC1 staining scan analysis across all samples. ∗*p* < 0.05, ∗∗*p* < 0.01, and ∗∗∗*p* < 0.001. *C*, IHC analysis of the relative level of CXXC5 expression was plotted against that of CUL4B, MTA1, and TSC1. *D*, CXXC5 expression level in 11 cases of breast carcinoma and adjacent normal breast tissues was analyzed by RT–qPCR. Each bar represents the mean ± SD from triplicate biological experiments. ∗*p* < 0.05, ∗∗*p* < 0.01, and ∗∗∗*p* < 0.001. *E*, bioinformatics analysis of The Cancer Genome Atlas dataset for the expression of CXXC5 in normal and primary tumor samples. *F*, the relationship between CXXC5 expression level and breast cancer patients’ survival time was predicted by Kaplan–Meier survival analysis (https://tcga-data.nci.nih.gov/docs/publications/tcga/). IHC, immunohistochemical; qPCR, quantitative PCR; TSC1, tuberous sclerosis complex subunit 1.

To further unravel the function of the CXXC5–CRL4B–NuRD complex in breast cancer development, we used immunohistochemical staining to detect the protein levels of CXXC5, CUL4B, MTA1, and TSC1 in two tissue arrays from breast cancer patients, which consisted of 21 pairs of breast carcinoma samples of grade II and paracancerous tissues, as well as 86 breast carcinoma samples, including grade I (9), II (51), or III (26), from patients with breast cancer. We detected significantly upregulated expression of CXXC5, CUL4B, or MTA1 in breast carcinoma samples compared with that in normal tissues (Fig. 7*A*), and their expression levels increased with the histological grades of the tumor specimens (Fig. 7*B*). Correspondingly, immunohistochemical staining showed that the expression of TSC1 in breast carcinoma specimens was lower than that in normal tissues, and the expression level decreased with increasing tumor histological grade (Fig. 7, *A* and *B*). In addition, the relative expression level of CXXC5 was positively correlated with that of CUL4B and MTA1 but negatively correlated with that of TSC1 (Fig. 7*C*). Next, we analyzed CXXC5 mRNA expression levels in breast carcinoma specimens with paired paracancerous tissues from 11 breast cancer patients using RT–qPCR. We detected a higher CXXC5 mRNA expression level in breast carcinomas than in adjacent tissues in seven of these paired samples (Fig. 7*D*). Moreover, The Cancer Genome Atlas analysis showed strikingly increased expression of CXXC5 in breast cancer samples (Fig. 7*E*). Finally, we further performed Kaplan–Meier survival analysis (http://kmplot.com/anaylsis/), which indicated that a high level of CXXC5 expression was significantly associated (*p* = 0.001) with a lower overall survival in breast cancer patients (Fig. 7*F*). Together, these data confirm that high expression of CXXC5 promotes breast cancer development.

## Discussion

In this study, we found that CXXC5 recruits the CRL4B complex and the NuRD (MTA1) complex, inhibits the expression of several genes, including *TSC1*, and indirectly activates downstream pathways, such as the mTOR signaling pathway. Previously, CXXC5 was reported to be involved in both repressing and activating gene transcription (7, 43). Whether these functions are cell type or context specific is currently unknown. In addition, whether CXXC5 interacts with distinct corepressor complexes to repress gene transcription also remains to be further investigated. Nevertheless, our results support a model in which CXXC5 recruits transcription repressors to inhibit target gene expression. However, as the CXXC domain is conserved among other CXXC family members, how to achieve the functional specificity of CXXC5 remains to be explored.

Epigenetic mechanisms contribute significantly to define the transcriptional landscape and the spatiotemporal patterns of gene expression. Owing to their great plasticity and elaboration, epigenetic mechanisms, including histone ubiquitination, methylation, and deacetylation, regulate tissue-specific patterns of gene expression and enable integration of various environmental stimuli to specify a transcriptional response. We demonstrated that in breast cancer cells, CXXC5 is responsible for initiating the transcriptional repression of *TSC1*, followed by monoubiquitination of H2AK119 by the CRL4B complex and NuRD (MTA1) complex–mediated histone deacetylation, which create a repressive chromatin environment. Interestingly, we found that NuRD-mediated histone deacetylation is dependent on CRL4B complex–directed H2AK119 monoubiquitination. The biological significance of this relationship remains unclear. Nevertheless, our results indicate that CXXC5 plays a crucial role in the complexity of epigenetic regulation, highlighting that the importance of CXXC5 in the process of tumorigenesis cannot be underestimated.

The regulation of the TSC1/mTOR pathway by the CXXC5–CRL4B–NuRD complex may have a significant physiological significance. Despite CXXC5 being deleted in leukemia, it is overexpressed in solid tumors (4, 5), which is consistent with our finding demonstrating the oncogenic potential of CXXC5. In addition, we also showed that CXXC5 is involved in tumor-immune regulation. Fighting cancer still remains a daunting task, and immunotherapy is a promising approach. New insights into the function of the host immune response in tumor development and the effects of different modalities of treatment have permitted the identification and discovery of the clinical use of novel immune checkpoint inhibitors for cancer therapy (44). Nonetheless, some patients do not benefit from immunotherapy and even experience relapse. Epigenomic signatures of immune cells and cancer cells appear to be accurate predictors of the outcomes of prognosis in patients undergoing immunotherapy. In recent years, the prognosis of cancer patients has improved owing to advances in immunotherapeutic approaches (45), such as anti-PD-1/PD-L1, which have shown effective clinical responses in patients with various cancer types (46). Despite this unprecedented efficacy, tumors often develop immune-escape mechanisms that hinder the success of immunotherapy and acquire resistance to immunotherapy (47, 48). Hence, determining the molecular mechanisms of drug resistance, accurate prognostic predictors, and novel molecules for combination immunotherapy is urgently needed. Fine-tuning the activity of the mTOR signaling pathway is required to maintain proper immune function. Interestingly, the antitumor effect of PD-L1 could rely on the regulation of the mTOR pathway (49). Since CXXC5 was highly expressed in some patients with solid tumors, and the expression level of CXXC5 was positively correlated with mTOR signaling pathway, our results demonstrate that targeting CXXC5 is likely to be an effective means of treating cancer patients, such as those receiving immunotherapy, as a biotherapeutic strategy.

Vitamins are organic nutrients required in small amounts for many biochemical reactions. Among them, vitamin B family members and vitamin C are water-soluble vitamins that can be absorbed *via* the small intestine. Lipid-soluble vitamins include vitamin A, D, E, and K. As vitamins are key cofactors for enzymes involved in epigenetic reprogramming and transcription regulation, which are known to be deregulated during oncogenic processes, they are being investigated as promising novel targets. However, it has also been reported that vitamins may promote carcinogenesis and metastasis, depending on the dose or form of administration (50). According to our model, the expression of CXXC5 was downregulated after vitamin D treatment and upregulated by vitamin B2 treatment. In addition, gain of function of CXXC5 induced inhibition of target genes, such as *TSC1*, and regulated the mTOR pathway positively, which could regulate the expression of PD-L1 in cancer cells (19). If our interpretation is correct, it is conceivable that vitamin B2 stimulation elevates CXXC5 expression, representing an effective complement to cancer immunotherapy and a strategy for breast cancer patients to achieve immunotherapy survival, whereas downregulation of CXXC5 leads to insensitivity to PD-1 blockade. However, the molecular mechanism underlying the relationship among CXXC5, immunotherapy, and vitamin supplementation in cancer patients remains to be further elucidated.

In summary, our data support that CXXC5 acts as a potential suppressor of TSC1 and indicates that the vitamin-CXXC5/CRL4B/NuRD-TSC1/mTOR-PD-L1 axis is associated with breast carcinogenesis, providing insights into the function of CXXC5 in tumorigenesis and supporting the use of CXXC5 as a diagnostic and therapeutic target for breast cancer in the future.

## Experimental procedures

### Antibodies and reagents

The antibodies used in this research were as follows: anti-MBD2/3 (catalog no.: sc-271521, for WB and IP), antimouse immunoglobulin G (catalog no.: sc-2025, for IP), and anti-CHD4 (catalog no.: sc-55606, for WB) from Santa Cruz Biotechnology; anti-HDAC1 (catalog no.: H3284; for WB and IP), anti-HDAC2 (catalog no.: H3159; for WB and IP), and FLAG (catalog no.: F3165) from Sigma; anti-rabbit immunoglobulin G ( catalog no.: PP64B; for IP) from Millipore; anti-CXXC5 (catalog no.: 51523; for WB and IP), anti-MTA1 (catalog no.: 9112; for WB and IP), anti-mTOR (catalog no.: 2983; for WB), anti-p-mTOR (catalog no.: 2971; for WB), anti-4EBP1 (catalog no.: 9644; for WB), anti-p-4EBP1 (catalog no.: 2855; for WB), anti-TSC1 (catalog no.: 6935; for WB), anti-S6K (catalog no.: 9202; for WB), and anti-p-S6K (catalog no.: 9205; for WB) from Cell Signaling Technology; anti-MTA2 (catalog no.: ab8106; for WB and IP), anti-ROC1 (catalog no.: ab2977; for WB and IP), anti-LC3B (catalog no.: ab192890; for WB and immunofluorescence), and anti-p62 (catalog no.: ab109012; for WB) from Abcam; anti-CXXC5 (catalog no.: 16513-1-AP; for ChIP and ChIP-Seq) and anti-CUL4B (catalog no.: 12916-1-AP; for WB and IP) from Proteintech; anti-DDB1 (catalog no.: GTX100130; for WB and IP) from GeneTex; and anti-RbAp46/48 (catalog no.: A6967; for WB and IP) from Abclonal. Anti-FLAG M2 affinity gel (catalog no.: A2220), anti-CUL4B (catalog no.: HPA011880; for ChIP-Seq), and puromycin (catalog no.: P8833) were purchased from Sigma.

### Plasmids

The FLAG-tagged *MTA1* in Taq2B or GST-tagged *CXXC5* in pGEX4T-3 vector and FLAG-tagged *CXXC5*, *CUL4B*, *DDB1*, *ROC1*, *RbAp46*, *RbAp48*, *MBD2/3*, *HDAC1*, or *HDAC2* in pcDNA3.1. Taq2B or pcDNA3.1 vector was purchased from YouBio.

### Cell culture

Cell lines were obtained from the American Type Culture Collection and cultured following the relevant guidelines. All experimental cells were verified to be free from mycoplasma contamination by morphological analysis and growth characteristic detection. Glucose starvation experiments were performed by rinsing cells with glucose-free Dulbecco's modified Eagle's medium (Gibco; catalog no.: 11966025), followed by continuous culture in glucose-free Dulbecco's modified Eagle's medium containing 10% fetal bovine serum.

### Immunopurification and silver staining

MCF-7 cells stably expressing FLAG-CXXC5 were cultured, and whole protein was extracted. The relevant immunoaffinity column was prepared using anti-FLAG M2 affinity gel following the manufacturer’s instructions. Approximately 5 × 10^7^ cells were lysed, followed by loading of the lysates onto a 1 ml bed, equilibrated the column to complete the binding of protein complexes to the column resin. FLAG peptide was used to elute FLAG-labeled proteins from the column. Eluted proteins were heat denatured and then electrophoresed using 4–12% Bis–Tris gel followed by silver staining of the gels with Pierce silver stain kit. Individual protein bands were excised, extracted, and analyzed by LC–MS/MS (Agilent; catalog no.: 6340) sequencing.

### IP

Cells were lysed using NETN buffer (150 mM NaCl, 2 mM EDTA, 50 mM Tris–HCl, pH 8.0, 0.2% Nonidet P-40) for 20 min at 4 °C, followed by centrifugation to collect the supernatant as the cell protein. About 500 μg of protein and control or specific antibodies (1–2 μg) was taken and incubated at 4 °C for 12 h, and then 50 μl of 50% protein G magnetic beads was added and incubated for another 2 h. After rinsing the beads five times with NETN buffer, the beads were resuspended in 2× SDS-PAGE loading buffer and boiled for 10 min to elute the precipitated proteins from the beads. Following SDS-PAGE analysis, immunoblotting was performed using the antibodies of interest.

### FPLC

A Superpose 6 size-exclusion chromatography column (GE Healthcare) equilibrated with DTT and calibrated with protein standards (Amersham Biosciences) was used. MCF-7 cell nuclear extracts were prepared and dialyzed against lysis buffer (20 mM Hepes [pH 8.0], 10% glycerol, 0.1 mM EDTA, 300 mM NaCl). Approximately 5 mg nuclear protein was concentrated to 0.5 ml using a Millipore Ultrafree centrifugal filter apparatus (3 kDa nominal molecular mass limit), then the concentrated nuclear proteins were applied to the column, which was eluted at a 0.5 ml/min flow rate, and then the flow through was collected separately.

### GST pull-down assays

The GST-tagged recombinant protein was expressed in BL21 *Escherichia coli.* Following the manufacturer’s recommendations, Promega's TNT Systems kit was used for *in vitro* transcription and translation of the target protein. Approximately 5 μg of the GST fusion protein, 30 μl of glutathione-Sepharose beads, and 5 to 8 μl of *in vitro* transcribed/translated products were reacted in binding buffer (75 mM NaCl, 50 mM Hepes, pH 7.9) in the presence of a protease inhibitor cocktail for 2 h at 4 °C for the GST pull-down assay. After multiple washings with binding buffer, the beads were resuspended in 30 μl of 2× SDS-PAGE loading buffer, and then the *in vitro* interaction of the relevant proteins was detected using WB.

### ChIP-Seq

Approximately 5 × 10^7^ cells were collected, and chromatin DNA was pulled with CXXC5, CUL4B, and MTA1 antibodies. The pulled down DNA was recovered using a Qiagen PCR purification kit. The purified DNA was sent to NOVOGENE for in-depth whole-genome DNA sequencing. Raw data were filtered using the Illumina analysis pipeline, aligned to the unmasked human reference genome (GRCh38 and hg38) using Bowtie2, and then analyzed using model-based analysis on ChIP-Seq MACS2. The genomic distribution of CXXC5-, CUL4B-, and MTA1-binding sequences was revealed using ChIPseeker. MEME (http://meme-suite.org/tools/meme) was used for analyzing the CXXC5, CUL4B, and MTA1 DNA-binding motifs. DeepTools (https://deeptools.readthedocs.io/) was used to form a bigwig trace heatmap for the genome browser for further visualization of ChIP-Seq data.

### qChIP and ChIP/Re-ChIP

Eluted DNA was recovered using a kit for PCR purification, and qChIP analysis was performed on the ABI 7500-FAST System or LightCycler 480 Real-Time PCR system by real-time RT–qPCR. The procedure of the Re-ChIP experiment was the same as that for the primary IP. Here, beads from the first IP were incubated with elution buffer containing DTT at a final concentration of 10 mM for 30 min at 37 °C; then, the mixture was diluted 1:50 in dilution buffer (1% Triton X-100, 2 mM EDTA, 150 mM NaCl, 20 mM Tris–HCl [pH 8.1]), followed by the addition of the secondary antibodies. Finally, the elution step was completed using 1% SDS solution in Tris–EDTA buffer (pH 8.0). Table S3 lists the qChIP qPCR primers used in this research.

### RNA interference

Cells were subjected to RNA interference using Lipofectamine RNAi MAX (Invitrogen) as per the manufacturer’s instructions. siRNA was solubilized at a working concentration of 10 nM, and the cells were harvested 72 h after transfection. For the RNAi experiment, no less than three independent siRNA sequences were selected for each gene, and the two with the highest knockdown efficiency were selected for use in subsequent studies. Table S4 lists the siRNA sequences used in this research.

### RT–qPCR

After total cellular RNA was extracted with TRIzol reagent (Invitrogen), it was reverse-transcribed into complementary DNA using a Reverse Transcription System (Roche). The transcription levels of related genes were quantitatively analyzed by RT–qPCR using the Power SYBR Green PCR Master Mix (Roche) and ABI PRISM 7500 sequence detection system or LightCycler 480 real-time PCR system. The transcript level of *GAPDH* was used as an internal reference. Table S5 lists the primers used in this research.

### Lentivirus production and infection

Recombinant lentiviruses expressing CXXC5, CUL4B, MTA1, shCXXC5, shCUL4B, shMTA1, or shTSC1 were prepared by Shanghai Gene Pharmaceutical Company. Approximately 5 × 10^5^ cells were infected with indicated viruses in a 60 mm dish using polybrene. The infected breast cancer cells were then classified depending on the expression of targets. Table S6 lists the shRNA sequences used in this research.

### Transmission electron microscopy

Cells were fixed with 2.5% glutaraldehyde at 4 °C for 4 h, washed five times with 0.1 M phosphate buffer, and postfixed with 1% osmic acid at room temperature and then fixed for 2 h. Samples were then stained with 1% microfiltered uranyl acetate, dehydrated in increasing concentrations of ethanol, permeabilized, and embedded in SPI-Pon 812 medium. Ultrathin sections were cut with a Leica Ultracut Microtome after the resin was polymerized for 48 h at 60 °C. Sections were stained with 1% uranyl acetate and 0.2% lead citrate, and images were acquired using a JEM-2100 electron microscope. Vesicles with double-membrane structures that engulf cytoplasmic material were defined as autophagosomes. A monolayer structure with significantly enlarged volume and abundant undegraded materials (autophagosomes and other membrane components) were defined as enlarged autolysosomes.

### Puromycin incorporation assay

MCF-7 cells stably expressing the expression constructs and specific shRNAs *via* lentivirus infection were incubated in puromycin (Sigma) for 30 min, harvested, rinsed twice with cold PBS, and lysed with NETN buffer in the presence of a protease inhibitor cocktail (Roche). Equal amounts of whole protein extracts were then analyzed by WB using the antipuromycin antibody. Coomassie blue staining was used to represent the total proteins. Statistical differences between groups were calculated from the scanning gray value.

### Colony formation assay

MCF-7 cells were stably transfected with the expression constructs and specific shRNA expression plasmids. These cells were cultured for 14 days and stained with crystal violet after fixation with 4% paraformaldehyde for 15 min for colony observation and counted using a light microscope. Each experiment was performed in triplicate and repeated at last three times.

### Tumor xenografts

MCF-7 cells were plated and infected *in vitro* with lentiviruses carrying control shRNA, CXXC5 shRNA, or CXXC5 shRNA + TSC1 shRNA, or lentiviruses carrying vector, CXXC5, CXXC5 + shCUL4B, or CXXC5 + shMTA1. Then, 48 h after infection, 3 × 10^6^ viable MCF-7 cells in 100 μl PBS were injected into 6- to 8-week-old ovariectomized female athymic mice (n = 5). E2 pellets (0.72 mg per pellet, 60-day release; Innovative Research of America) were implanted 1 day before tumor cell injection. Tumor growth was monitored over the following 4 weeks. Tumors were measured weekly using a Vernier caliper. The volume was calculated according to the formula: π/6 × length × width (2).

### Tissue specimens

Carcinoma and paracancerous specimens were obtained from breast cancer patients with available data of clinicopathological characteristics. Immediately following surgical excision, tissues were placed in liquid nitrogen and kept at −80 °C until mRNA and protein extraction. Breast carcinoma and paracancerous tissue arrays were prepared, followed by staining for immunohistochemical analysis using standard 3,3′-diaminobenzidine staining protocols.

### Statistics

Data from biological triplicate experiments are presented with error bars as the mean ± SD. Two-tailed *t* test and ANOVA were used to compare the different groups of data. Statistical significance was set at *p* < 0.05. All the statistical testing results were determined by SPSS software (Statistical Product and Service Solutions). Data for Kaplan–Meier survival analysis were from http://kmplot.com.

### Study approval

All animal handling and experiments were approved by the Animal Care Committee of Capital Medical University. The collection and experimental analysis of patient tissue samples were approved by the Ethics Committee of the Capital Medical University, and informed consent was obtained from all patients.

## Data availability

All results required to support the conclusions are presented in the article and/or the supporting information. The ChIP-Seq data can be found in the Gene Expression Omnibus database (accession number: GSE205362). Additional data relevant to this article are available from the authors upon reasonable request. Kaplan–Meier survival analysis of the relationship between survival time and CXXC5 expression levels in patients with breast cancer was conducted using packages from https://tcga-data.nci.nih.gov/docs/publications/tcga/.

## Supporting information

This article contains supporting information (13, 14, 15, 32, 34, 35).

## Conflict of interest

The authors declare that they have no conflicts of interest with the contents of this article.
