# Supplementary material for: Zinc-finger protein CXXC5 promotes breast carcinogenesis by regulating the TSC1/mTOR signaling pathway
Source: J Biol Chem. 2022 Dec 17;299(1):102812. doi: 10.1016/j.jbc.2022.102812 (PMC9860500; doi:10.1016/j.jbc.2022.102812)
Supplement: Table S3 [file mmc3.docx]

**Supplemental Table 3. siRNA sequences**

| siRNAs | Sequences |
| --- | --- |
| CXXC5-1 | CCGUCUUUAGAACCAAAAATT |
| CXXC5-2 | CGUCUUUAGAACCAAAAAUTT |
| CUL4B-1 | GAAUGUUCCGGGAAAUAUUTT |
| CUL4B-2 | GUGUACAACCAGUUGAAAUTT |
| MTA1-1 | GCAUCUUGUUGGACAUAUUTT |
| MTA1-2 | CCGAGUCGCUCAAGUCCUATT |
| TSC1-1 | GUGACACUAUGGUAACCAATT |
| TSC1-2 | CGUUCUCAUUACAGUAUGATT |

**Supplemental Table 4. Lentiviral shRNA sequences**

| shRNAs | Sequences |
| --- | --- |
| Control | 5'-GCTCGCCTGTCTACTAACTAA-3' |
| CXXC5 | 5‘-GCTCTGGAGAAGGTGATGCTT-3 |
| CUL4B | 5'-AATATTTCCCGGAACATTCTG-3' |
| MTA1 | 5‘-GGCTAACTTATTCCGAGAATG-3’ |
| TSC1 | 5‘-GCGAATTCATCCGGAATTAGT-3’ |

**Supplemental Table 5. RT-qPCR primers**

| **Genes** | **Forward Primer Sequences** | **Reverse Primer Sequences** |
| --- | --- | --- |
| *CXXC5* | CCGAGCGTCGGAACAAGAG | CCACTGCTGCCAAAAGAAGAG |
| *CUL4B* | CAAACGGCCTAGCCAAATCTT | CAGTTTTTGCCAGGTTTCATCTG |
| *MTA1* | ACGCAACCCTGTCAGTCTG | GGGCAGGTCCACCATTTCC |
| *TSC1* | CAACAAGCAAATGTCGGGGAG | CATAGGGCCACGGTCAGAA |
| *GSK3B* | AGACGCTCCCTGTGATTTATGT | CCGATGGCAGATTCCAAAGG |
| *FAS* | AGATTGTGTGATGAAGGACATGG | TGTTGCTGGTGAGTGTGCATT |
| *RNF152* | CTGTCATCGCCATTCCACACA | GCAGCATGTAGCACCCATTG |
| *PD-L1* | TGGCATTTGCTGAACGCATTT | TGCAGCCAGGTCTAATTGTTTT |
| *GAPDH* | GAAGGTGAAGGTCGGAGTC | GAAGATGGTGATGGGATTTC |

**Supplemental Table 6. qChIP primers**

| **Genes** | **Forward Primer Sequences** | **Reverse Primer Sequences** |
| --- | --- | --- |
| *TSC1* | ACAGTCCCTCCAGCCTACA | CCGTCTATCCTTCCTTTCG |
| *GSK3B* | GCCCGTGGTAGCTTTGGAT | GAACAATGGACGCTGGTCTTAT |
| *FAS* | TCGGTGCTGACTTATTTCCTAC | CTGACAAGCCAAGCCAAAG |
| *RNF152* | CTGCCCTTTCCCGCTTCC | GGTCAGTGAGTTTCGGTTTGTT |
