# Supplementary material for: Zinc-finger protein CXXC5 promotes breast carcinogenesis by regulating the TSC1/mTOR signaling pathway
Source: J Biol Chem. 2022 Dec 17;299(1):102812. doi: 10.1016/j.jbc.2022.102812 (PMC9860500; doi:10.1016/j.jbc.2022.102812)
Supplement: Table S4 [file mmc4.docx]

**Supplemental Figures**


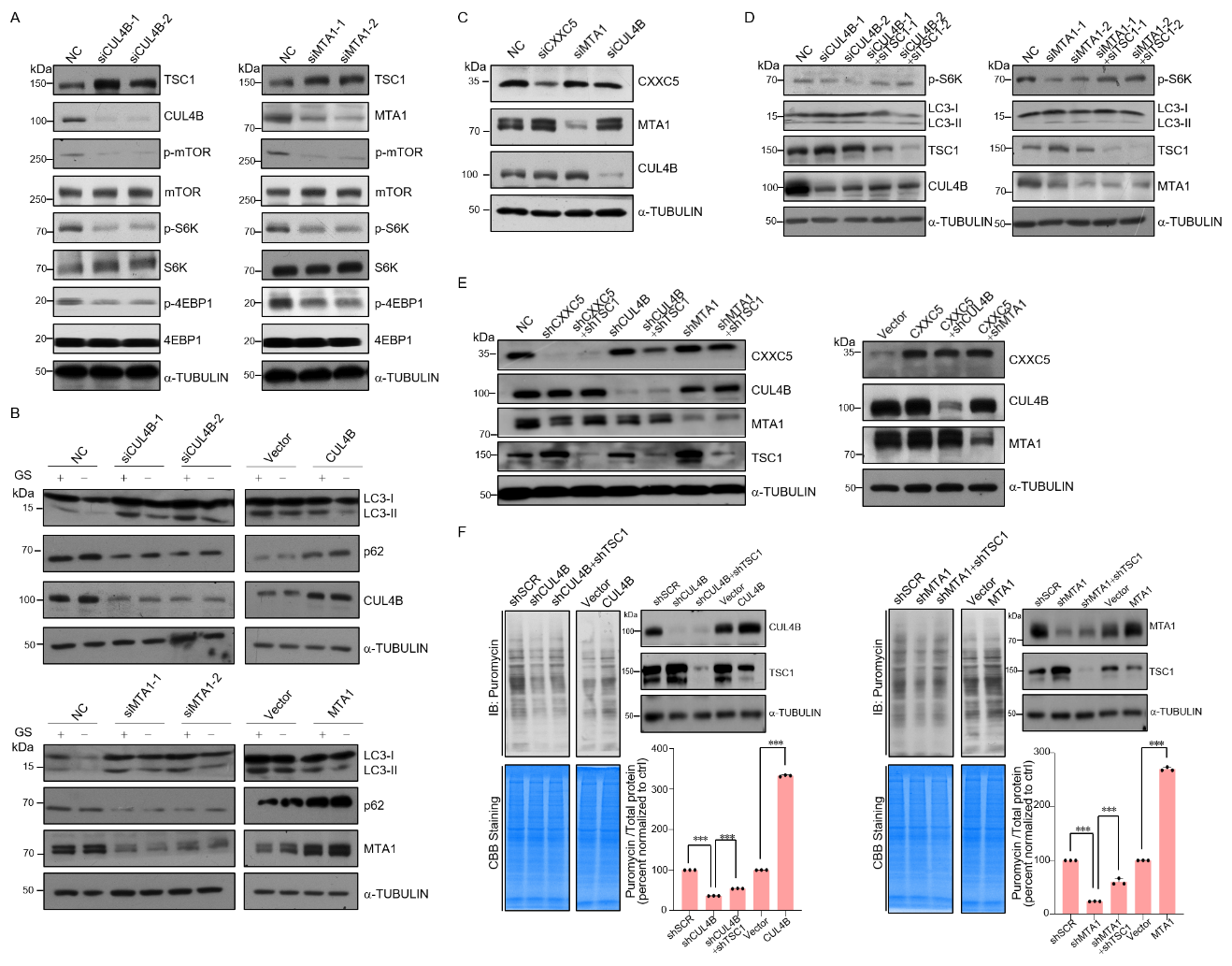


**Figure S1, related to Figure 4. CXXC5 regulates mTOR signaling and impacts autophagy and protein synthesis by transcriptional repression of *TSC1*.** (A) After transfection of different groups of CUL4B and MTA1 siRNAs into MCF-7 cells, western blotting was used to detect the indicated protein expression levels. (B) The indicated expression constructs or specific siRNAs were transfected into MCF-7 cells; the transfected cells were treated with or without 4-h glucose starvation (GS) and then the expression levels of the indicated proteins were analyzed using western blotting. (C) CXXC5, CUL4B, or MTA1 siRNAs were transfected into MCF-7 cells. The knockdown efficiencies of CXXC5, CUL4B, and MTA1 were verified by western blotting. (D) The MCF-7 cells transfected with the indicated siRNAs were assayed for autophagy markers by western blotting. (E) MCF-7 cells were infected with the specific expression constructs and indicated shRNA lentiviruses. The efficiency of knockdown or overexpression was verified by western blotting. (F) MCF-7 cells stably expressing the specific expression constructs and indicated shRNA lentiviruses were assessed for their ability for *de novo* protein synthesis using anti-puromycin immunoblotting and pulsed with a final concentration of 1 μM puromycin for 30 min. Coomassie blue staining represented the total proteins.

**References**

1. Dibble, C. C. *et al.* TBC1D7 is a third subunit of the TSC1-TSC2 complex upstream of mTORC1. *Mol Cell* **47**, 535-546, doi:10.1016/j.molcel.2012.06.009 (2012).
2. Lee, D. F. *et al.* IKK beta suppression of TSC1 links inflammation and tumor angiogenesis via the mTOR pathway. *Cell* **130**, 440-455, doi:10.1016/j.cell.2007.05.058 (2007).
3. Inoki, K., Corradetti, M. N. & Guan, K. L. Dysregulation of the TSC-mTOR pathway in human disease. *Nat Genet* **37**, 19-24, doi:10.1038/ng1494 (2005).
4. Mallela, K. & Kumar, A. Role of TSC1 in physiology and diseases. *Mol Cell Biochem* **476**, 2269-2282, doi:10.1007/s11010-021-04088-3 (2021).
5. Gomez-Sanchez, R. *et al.* mRNA and protein dataset of autophagy markers (LC3 and p62) in several cell lines. *Data Brief* **7**, 641-647, doi:10.1016/j.dib.2016.02.085 (2016).
6. Ma, X. M. & Blenis, J. Molecular mechanisms of mTOR-mediated translational control. *Nat Rev Mol Cell Biol* **10**, 307-318, doi:10.1038/nrm2672 (2009).
